# Aging with intellectual disabilities: a study in Portugal about clinical conditions, functionality, and frailty

**DOI:** 10.3389/fnhum.2026.1952261

**Published:** 2026-10-14

**Authors:** Mariana Letra, Joao Tavares, Sara Guerra, Ana Rita Peralta, Fatima Duarte, Raquel Amaral, Liliana Sousa

**Affiliations:** 1 RISE-Health, University of Aveiro, Aveiro, Portugal; 2 Federação Nacional de Cooperativas de Solidariedade Social, Lisboa, Portugal; 3 CERCI Lisboa, Lisboa, Portugal; 4 University of Aveiro, Aveiro, Portugal

**Keywords:** clinical status conditions, frailty, functionality, healthy aging, intellectual disabilities, malnutrition risk

## Abstract

The life expectancy of people with intellectual disabilities (ID) has increased more than that of the general population, demanding a deeper understanding of their health profiles to inform tailored healthy aging interventions. This study aimed to characterize persons with ID aged ≥ 45 years in terms of clinical conditions (degree of ID, comorbidities, and medication), malnutrition risk, functional capacity and frailty. To our knowledge, this is the first study to address this topic in Portugal. A cross-sectional, descriptive-correlational design was used, with a non-probability convenience sampling of 234 individuals with ID aged ≥ 45 years. Questionnaires were completed by organizational mediators, achieving a response rate of 18.5%. The mean age was 53.32 years; 56.4% were male, 97.9% were single, and 98.3% had no children. The main findings are as follows: (i) degree of ID: 52.1% moderate, 25.6% severe, 15% mild and 7.3% profound; (ii) high prevalence of comorbidities; (iii) 38.9% were on five or more medicines (polypharmacy); (iv) 44.9% were at risk of malnutrition and 4.7% were malnourished; (v) 23.1% had total or severe dependence in basic activities of daily living; (vi) 85.5% were severely and 14.5% were moderately dependent in instrumental activities of daily living; and (vii) 46.3% were classified as being frail. Older adults with ID seemed to present complex clinical conditions that required a higher intensity of support. These findings contribute to the continuous development of a model of care tailored to older people with ID, particularly healthy aging interventions.

## Introduction

Intellectual disability (ID) refers to a group of developmental conditions characterized by a significant impairment of cognitive functions associated with limitations in learning, adaptive behavior and skills (World Health Organization, 2017). The life expectancy of people with ID has increased more than that of the general population (Takenoshita et al., 2023; Wissing et al., 2022). Overall, their life expectancy is now comparable with that of the general population, except for a shorter life expectancy for people with more severe intellectual and multiple disabilities and people with Down syndrome (Wissing et al., 2022). Although there is still no consensus regarding the chronological age at which a person with ID is considered an older adult, the World Health Organization (2000) proposed the age of 50 years as a benchmark. However, the literature also recommends that planning for specific support should begin from the age of 45 onwards (Buys et al., 2012; Doyle et al., 2022; Hilgenkamp et al., 2011). Therefore, in the current study, we focused on individuals aged ≥45 years old.

The increased longevity of people with ID is a welcome and relevant demographic shift that comprises new challenges in terms of support and care (Sheerin et al., 2025). For instance, the proportion of older adults with ID in social services is increasing, with an age-related complexity that demands adjustments for the provision of care (Sheerin et al., 2023). Letra et al.'s (2025) recent study in Portugal involving 31 organizations supporting persons with ID showed that the number of people with ID aged ≥45 years increased more than five-fold between 2008 (312) and 2024 (1,731 people).

Therefore, providing healthy aging interventions for older adults with ID is essential. The World Health Organization (2021) has defined healthy aging as a process of maintaining functional ability to enable wellbeing in older age. This is a broad definition that demands operationalization for specific older populations. A recent scoping review focused on healthy aging for older adults with ID (El Mrayyan et al., 2025) has shown different interrelated definitions, such as maintaining functional ability and well-being (Santos et al., 2022); enabling people to live in accordance with their preferences and make independent choices (Johansson et al., 2017); and supporting health promotion, developing formal policies and supporting community-based health and participation (Spassiani et al., 2019). Having a definition of healthy aging for older adults with ID is a need that will support intervention development. Currently, the literature indicates that healthy aging interventions for people with ID are scarce, incipient and sporadic (El Mrayyan et al., 2025; Santos et al., 2022). In addition, this population has a range of unmet health and social care needs associated with a lack of knowledge about their (complex) aging (Bigby et al., 2015; Sheerin et al., 2025). In brief, increasing healthy aging interventions for this population group demands a better conceptualization of healthy aging, along with more knowledge about this group's clinical condition, functionality and frailty (El Mrayyan et al., 2025; Sheerin et al., 2025).

In terms of physical health, older adults with ID present a high incidence of obesity, hypertension, type 2 diabetes mellitus, cardiovascular disease and respiratory problems (Dean et al., 2021). Airway obstruction is also a serious and underestimated condition, with consequences for psychological health and the potential to be fatal (Manduchi et al., 2020). This group is at a higher risk of malnutrition compared with the general population (Ball et al., 2012; Bertoli et al., 2006; Gast et al., 2022) due to multiple factors (e.g., swallowing difficulties, polypharmacy, cognitive impairments, difficulties with independence and reduced access to adequate healthcare). Obesity carries a higher risk of developing chronic conditions, such as diabetes and cardiovascular disease. The literature also highlights the association between oral health and nutritional status (Islamoglu et al., 2024). People with ID have a higher prevalence of oral health conditions (e.g., untreated caries and periodontal disease) than the general population (Morgan et al., 2012). Consequently, oral health problems increase the risk of malnutrition and chronic oral diseases (Ziegler and Spivack, 2018).

Regarding mental health, depression and anxiety are the most prevalent problems among older adults with ID compared with younger adults with ID and the general population (Bond et al., 2020; Hussain et al., 2021; O'Dwyer et al., 2017; Timmins et al., 2023). People with ID are at a higher risk of developing early-onset dementia (MacHale et al., 2024). People with ID are more likely to be prescribed psychotropic medications and to be on multiple psychotropic medications. In a sample of 753 individuals with ID aged 41–90 years, O'Dwyer et al. (2017) reported that 59.1% were taking some form of psychotropic medication, the most common being antipsychotics; of these, 66.2% were on multiple psychotropic medications. Psychotropic polypharmacy in older adults with ID is associated with living in residential care, a higher prevalence of mental health conditions (controversial) treatment for challenging behaviors and treatment for sleep problems (Bond et al., 2020; O'Dwyer et al., 2017).

In addition to chronic conditions, geriatric syndromes are similarly common in older adults with ID, particularly frailty syndrome (Grohmann et al., 2024). This syndrome is characterized by a decline in strength, endurance and physiological functions (Grohmann et al., 2024; Morley et al., 2013). It represents a state of vulnerability to adverse events (e.g., illness, hospitalization and new medications; Kim and Rockwood, 2024), usually with early onset in people with ID (Evenhuis et al., 2012). A recent scoping review has indicated that the prevalence of frailty in older people with ID ranged from 9% to 84%, with this variation being explained by the definition of frailty used, the type of measurement tool and the degree of ID (Grohmann et al., 2024). Thus, the increase in life expectancy among people with ID in recent decades has been reflected in the increased incidence of frailty, which occurs alongside the disability and health problems experienced since childhood (Celebańska et al., 2022; Coppus, 2013; Evenhuis et al., 2012).

Functional capacity—that is, basic activities of daily living (BADLs) and instrumental activities of daily living (IADLs)—declines with age in people with ID and in the general population (Hilgenkamp et al., 2011; King et al., 2017). It tends to decline in the presence of the following: poorer health status, severe ID, reduced mobility, diminished or impaired cognitive ability and reduced sensory abilities (Delgado-Lobete et al., 2021; Heine et al., 2019; King et al., 2017). Performance in BADL is determined primarily by mobility, while performance in IADLs is more strongly determined by the severity of ID (Hilgenkamp et al., 2011). Thus, the decline in performance in BADL and IADL among people with ID tends to begin at an earlier age than in the general population (Delgado-Lobete et al., 2021; Hilgenkamp et al., 2011). Older adults with ID can be expected to require more support with activities of daily living (ADLs) as they age (King et al., 2017). A previous study (McCarron et al., 2019) indicated that 30% of people did not require support; around 35% had needs in four or more ADLs; and around 94% required at least some support with all ADLs.

This study aims to contribute to fulfilling the still scarce knowledge about this group by characterizing persons with ID aged ≥ 45 years old in terms of clinical conditions (e.g., degree of ID, comorbidities and medication), malnutrition risk, functionality (i.e., performance in BADL and IADL) and frailty. To our knowledge, this is the first study to address this topic in Portugal. Given the rapid growth of this population in Portuguese services and the absence of a systematic characterization of their profiles, the findings will provide an empirical basis for the development of targeted healthy aging interventions for older adults with ID.

## Methods

This was a cross-sectional, descriptive-correlational study, part of a larger project that aimed to identify the needs of a social organization to respond to the aging of clients with ID. This project was approved by the Ethics Committee of the Health Sciences Research Unit: Nursing (Opinion No. P1049_06_2024).

### Context and sample

This study was carried out in organizations affiliated with or similar to FENACERCI (the Portuguese National Federation of Social Solidarity Cooperatives). A non-probability convenience sampling procedure was used. Each participating organization identified clients who met the inclusion criteria: (i) having a diagnosis of ID and (ii) being aged ≥45 years. In total, 1,308 eligible clients, responses were received from 242 clients, corresponding to a response rate of 18.5%. Of these, eight were excluded due to an excessive number of missing values. The final analytical sample comprised 234 individuals with intellectual disability aged 45 years or older.

### Data collection procedure

Data collection was launched by FENACERCI via its institutional mailing list. The invitation email was sent to all 57 associate organizations and six similar organizations. The message included information for the participants, with a detailed description of the study, objectives and instructions on how to access and complete the questionnaire; a link to the online questionnaire (hosted on the LimeSurvey platform); a PDF version of the questionnaire for prior review; templates for the informed consent forms (for the respondent mediator, the legal representative of the person with ID aged ≥ 45 years, and the person with ID); and the research team's contacts. The email was sent to the administration of the organizations, asking them to designate a person (mediator) in the organization to carry out the data collection. When the administration's designated person agreed to collaborate, the person would sign the informed consent form and contact the first author for further clarification. This person (mediator) would identify the clients fulfilling the inclusion criteria, contact their legal representative and explain the study and the collaboration asked (information to the participants sent in the email) and obtain their informed consent (sent in the email). The legal representative would then explain the study to the person with ID and obtain their assent. This consent granted authority to the mediator to consult the client process and complete the questionnaire. The research team never had access to this process. Data collection was carried out between 2 June and 31 October 2025. During this period, six reminders were sent to boost the response rate. The research team ensured individualized support through direct contact and by answering queries throughout the data collection period. All queries submitted to the research team—via email or telephone—were promptly addressed, ensuring continuous support for the participating organizations and the reliability of the data collection process.

*Instrument*. The instrument was developed for this study based on (i) a review of the literature on biopsychosocial changes associated with aging in the population with ID (e.g., Boyd and Baril, 2018; Lynch et al., 2025; Wallace and Scheepers, 2018); (ii) a search for validated instruments for the older Portuguese population with ID, which revealed that no such instruments were available; and (iii) a collaborative process between the research team at the University of Aveiro, which has experience in assessing the needs of older adults, and the FENACERCI team, which has in-depth knowledge of the profile of older adults with ID. At the end of this process, a preliminary version of the instrument was produced and subsequently pre-tested with two organizations selected randomly by the research team between 9 December 2024 and 7 February 2025. Each organization provided information on two clients meeting the inclusion criteria. The pre-test involved completing the questionnaire and, at the end, answering the following questions: (i) administration (access to the platform, clarity of information provided to participants and difficulties), (ii) design (suitability of layout and font size), (iii) clarity and comprehensibility of content (coherence and logic of questions and response options, logic and interconnection between questions), (iv) acceptability (suitability and relevance), (v) completion (time taken to complete) and (vi) overall assessment (global opinion). The results indicated that the questionnaire took 30–45 min to complete and that it was considered clear, easy to complete and appropriate to the study objectives. Improvement was suggested for a lack of clarity in the response options regarding malnutrition risk. The phrase “no neuropsychological problems” needed to be clarified; thus, the following explanation was added: “no diagnosis of dementia.” The final instrument comprised the following variables:

*Sociodemographic data of the mediator*. These data comprise the role within the organization, age group, years working in the organization and years working with people with ID.

*Sociodemographic data of the persons with ID*. These data include the following variables: age, gender, marital status, number of children, household composition (who they live with), ability to sign (Yes/No), ability to read and write (Yes/No), educational qualifications, employment status, and main source of income.

*Clinical conditions*. This includes degree of ID classified according to the DSM-5-TR (American Psychiatric Association, 2022), presence of any other comorbid diagnoses, diagnosed medical conditions (lasting > 3 months), number of different prescribed medicines taken per day (1–4 medicines: not polymedicated, ≥5: polymedicated; Pedroso-Remelhe et al., 2022) and use of healthcare services in the last year (hospital admission, emergency services and family doctor).

*Malnutrition risk*. The Mini Nutritional Assessment-Short Form (MNA-SF^®^) was applied. The MNA-SF^®^ was developed by Guigoz et al. (1994) with the support of the Nestlé Nutrition Institute and validated for the Portuguese population by Loureiro (2008). It consists of six questions: (i) reduction in food intake over the past 3 months, (ii) weight loss over the past 3 months, (iii) mobility, (iv) whether there has been any psychological stress or acute disease during the past 3 months, (v) whether there are any neuropsychological problems such as depression or dementia and (vi) body mass index (BMI). The screening score ranges from 0 to 14 points, with the following cut-offs: 0–7, malnourished; 8–11, at risk of malnutrition; and 12–14, normal nutritional status (Loureiro, 2008). The MNA-SF^®^ has high sensitivity (93%) and specificity (88%) and is considered the gold standard (clinical assessment), with an inter-rater agreement of 0.74 (Loureiro, 2008). It has been validated for use in older people with ID, demonstrating good reliability, and its use by carers is recommended (Bastiaanse et al., 2012). The data enabled the calculation of the BMI, which was classified in accordance with the World Health Organization (2023): < 18.5, underweight; 18.5–24.9, normal weight; 25.0–29.9, overweight; and ≥30, obese.

*Functionality*. The Barthel Index was used to assess BADLs. Developed by Mahoney and Barthel (1965) and validated for the older Portuguese population by Araújo et al. (2007), the Barthel Index assesses the ability to perform BADLs: (1) Feeding (10, Independent; 5, Needs some help; 0, Dependent); (2) Transfers (15, Independent; 10, Needs some help; 5, Needs help from another person; 0, Dependent); (3) Toileting (personal hygiene) (5, Independent in shaving, washing face, brushing teeth; 0, Dependent); (4) Toilet use (10, Independent; 5, With help; 0, Dependent); (5) Bathing (5, Independent; 0, Dependent); (6) Mobility (15, Independent; 10, With some help; 5, Independent in a wheelchair; 0, Immobile); (7) Climbing and descending stairs (10, Independent; 5, Needs help; 0, Dependent); (8) Dressing (10, Independent; 5, Needs help; 0, Dependent); (9) Bowel control (10, Continent; 5, Occasional accidents; 0, Incontinent); (10) Urinary control (10, Continent; 5, Occasional accidents; 0, Incontinent). The total score ranges from 0 to 100 points, with higher scores indicating greater independence. The categories are independent (100), mild dependence (91–99), moderate dependence (61–90), severe dependence (21–60), and total dependence (0–20). The criterion validity of the Barthel Index in relation to the Lawton and Brody Index showed a strong positive correlation (*r* = 0.84). Reliability, assessed through internal consistency using Cronbach's alpha, was excellent (α = 0.96) (Araújo et al., 2007). The Barthel Index has been applied to older people with ID, recognizing that some of the dependency may be inherent to the underlying condition and not acquired during aging (Bastiaanse et al., 2012; de Leeuw et al., 2022; Hilgenkamp et al., 2011).

*The Lawton and Brody Index was used to assess IADLs*. Developed by Lawton and Brody (1969) and validated for the older Portuguese population by Sequeira (2007), the Lawton and Brody Index assesses the ability to perform IADLs. It comprises eight tasks with the following scores: (1) Housekeeping (1, Takes care of the house without help; 2, Does everything except heavy work; 3, Only performs light tasks; 4, Needs help with all tasks; 5, Unable to perform any tasks); (2) Doing the laundry (1, Does their own laundry; 2, Only washes small items; 3, Is unable to do the laundry); (3) Food preparation (1, Plans, prepares and serves without help; 2, Prepares if given the ingredients; 3, Prepares ready-made meals; 4, Unable to prepare meals); (4) Shopping (1, Shops independently; 2, Only does little shopping; 3, Goes shopping accompanied; 4, Unable); (5) Use of the telephone (1, Uses it without difficulty; 2, Dials a few familiar numbers; 3, Needs help; 4, Unable); (6) Transportation (1, Travels by public transport or drives own car; 2, Only travels via taxi; 3, Needs to be accompanied; 4, Unable); (7) Finances (1, Manages financial matters independently; 2, Manages day-to-day purchases; 3, Unable); (8) Medication (1, Responsible for taking medication; 2, Responsible if medication is prepared; 3, Unable). The final score is calculated by summing the scores for each IADL, and it ranges from 8 to 30 as follows: 8, independent; 9–20, moderately dependent; >20, severely dependent. Both the original and Portuguese versions showed high internal consistency, with Cronbach's alpha coefficients of 0.96 (Lawton and Brody, 1969) and 0.94 (Sequeira, 2007). This instrument has previously been used to assess the functional ability of older adults with ID, demonstrating good acceptability and applicability (Hilgenkamp et al., 2011; Tena-Bernal et al., 2021).

*Frailty*. The Clinical Frailty Scale (CFS v2.0) was developed by Rockwood et al. (2005) and validated for the older Portuguese population by (Pinto et al. 2021). This scale is based on clinical judgement to assess overall function and the degree of clinical frailty. Version 2.0 Pinto et al., (2021) comprises nine categories (Rockwood and Theou, 2020), ranging from “very good fit” to “terminally ill,” presenting the items in both visual and written form to assist the classification. The cut-off point is category five, with the following interpretation: 1–3, not frail; 4, pre-frail; and 5–9, frail (Pinto et al., 2021). Validity data show a strong correlation with the Tilburg Frailty Indicator scale (r = 0.683) and excellent test-retest reliability (ICC = 0.999), demonstrating good sensitivity, accuracy and high specificity. Various studies involving older adults with ID have used this scale (Festen et al., 2021; Grohmann et al., 2024).

### Data analysis

The data were analyzed using descriptive statistics, employing various parameters to characterize the distribution of the variables: frequency and percentage for categorical variables and mean (M) and standard deviation (SD) for continuous variables. In the inferential analysis, the chi-square test was used to assess the association between the sociodemographic, clinical and functional variables (including degree of ID) and frailty (frail vs. non-frail). For 2 × 2 contingency tables, the odds ratio (OR) with 95% confidence interval (CI) was calculated as the effect size, oriented so that OR > 1 indicates greater odds of frailty. For contingency tables with more than two categories in either variable, Cramér's V was computed as the effect size. For continuous variables, non-parametric Mann–Whitney tests were applied (following visual inspection of the Q–Q plots and the Kolmogorov–Smirnov test, *p* < 0.05). The rank-biserial correlation coefficient (*r*) with 95% CI was computed as the standardized effect size for the Mann–Whitney tests. All data were analyzed using IBM SPSS Statistics software version 30.0, with complementary effect size and confidence interval calculations performed in JASP version 0.18.3, maintaining a significance level of *p* < 0.05.

## Results

The questionnaire was completed by 32 mediators holding the following positions within the organizations: 23 technical coordinators (71.9%) and 9 superior technicians (28.1%). In terms of age groups, the following was observed: 14 (43.8%) were aged 40–49, nine were aged 50–59 (28.1%), four were aged 60–69 (12.5%), three were aged 18–29 (9.4%), and two were aged 30–39 (6.3%). The average length of service in the organization was 17.22 years (SD = 12.76). The mean number of years of working with people with ID was 17.84 years (SD = 12.36).

### Sociodemographic characteristics of people with ID

The sample consisted of 234 people with ID aged ≥ 45 years, with a mean age of 53.32 years, ranging from 45 to 77 years. Among them, 56.4% were male, 97.9% were single, and 98.3% had no children (Table 1). These individuals lived mainly with family members (52.6%), while 39.3% lived in social care settings, with 80 (34.2%) in a residential home, six (2.6%) in a residence for independence and inclusion and four (1.7%) in a residential care home for older adults. In terms of educational attainment, 66.2% could not read or write, 34.2% did not complete primary school, and 2.1% completed 12 years of schooling. The majority (89.3%) were pensioners or retired. Their main source of income was social security benefits (76.9%), followed by family income (12.8%). The participants had been supported by organizations for 30.82 years (12.52; 1–59) and by the current organization for 26.32 years (13.25; 1–51).

**Table 1:** Sociodemographic characteristics of people with ID ≥ 45 years (*n* = 234).

Variable	*n* (%)
**Sex**	
Female	102 (43.6)
Male	132 (56.4)
**Marital status**	
Single	229 (97.9)
Married/partnered	4 (1.7)
Divorced/separated	1 (0.4)
**Children**	
Yes	4 (1.7)
No	230 (98.3)
**Living with**	
Relatives	123 (52.6)
In an institution	92 (39.3)
Alone	9 (3.8)
Others/not relatives	6 (2.6)
Rotation	3 (1.3)
Couple	1 (0.4)
**Can read and write**	
Yes	79 (33.8)
No	155 (66.2)
**Years of formal education**	
Never attended school	66 (28.2)
< 4 years	80 (34.2)
4 years	36 (15.4)
5–6 years	21 (9)
7–9 years	15 (6.4)
10–12 years	5 (2.1)
Other	11 (4.7)
**Employment status** ^*^	
Pensioner/Retired	209 (89.3)
Unemployed	13 (5.6)
Supported employment	7 (3.0)
Employed (full-time)	1 (0.4)
Other	3 (1.3)
**Main source of income**	
Social benefits	180 (76.9)
Family income	30 (12.8)
Pensions	13 (5.6)
Work	9 (3.8)
Other	2 (0.8)
Variable	***M*** **(**±***DP*****) min-max**
Age	53.32 (6.44) 45–77
Number of children	0.02 (0.17) 0–2
Years being supported by organizations (*n* = 162)	30.82 (12.52; 1–59)
Years being supported by the current organization (*n* = 234)	26.32 (13.25; 1–51)

^*^1 missing value; M, mean; SD, standard deviation; min, minimum; max, maximum; CACI, Center for Activities and Training for Inclusion.

### Clinical conditions

Regarding the degree of ID, the results indicate the following: 122 (52.1%)—moderate, 60 (25.6%)—severe, 35 (15%)—mild, and 17 (7.3%)—profound (Table 2). The other most common diagnoses were epilepsy (*n* = 53; 22.6%), dementia (*n* = 53; 22.6%), impulse control disorder (*n* = 46; 19.7%), anxiety disorder (*n* = 36; 15.4%), cerebral palsy (*n* = 23; 9.8%), trisomy 21 (*n* = 17; 7.3%) and depressive and bipolar disorder (*n* = 13; 5.6%). In addition, 71 participants (30.3%) reported other chronic conditions (lasting more than 3 months): high blood pressure (*n* = 17; 23.94%), heart problems (*n* = 11; 15.49%), type 2 diabetes (*n* = 11; 15.49%), cancer (*n* = 6; 8.45%), respiratory problems (*n* = 5; 7.04%), high cholesterol (*n* = 5; 7.04%), obesity or being overweight (*n* = 5; 7.04%), thyroid problems (*n* = 4; 5.63%), anemia (*n* = 3; 4.23%), lupus (*n* = 1; 1.41%), rheumatoid arthritis (*n* = 1; 1.41%), constipation (*n* = 1; 1.41%), asthma (*n* = 1; 1.41%), arthritis (*n* = 1; 1.41%), seizures (*n* = 1; 1.41%), kidney failure (*n* = 1; 1.41%) and osteoporosis (*n* = 1; 1.41%). Regarding the number of different prescribed medicines that the participants take each day, 37 participants (15.8%) did not take any medication, 136 (58.1%) took 1–4 medicines, and 61 (26.1%) took ≥5 medicines (polypharmacy). In the past year, 91.3% of the participants were not hospitalized, 77.3% did not visit the emergency service, and 26.6% did not consult their family doctor.

**Table 2:** Clinical conditions (*n* = 234).

Variable	*n* (%)
**Degree of ID**	
Mild	35 (15)
Moderate	122 (52.1)
Severe	60 (25.6)
Profound	17 (7.3)
**Other diagnosis**	
Epilepsy	53 (22.6)
Dementia	53 (22.6)
Impulse control disorder	46 (19.7)
Anxiety disorder	36 (15.4)
Cerebral palsy	23 (9.8)
Trisomy 21	17 (7.3)
Depressive and bipolar disorder	13 (5.6)
Schizophrenia spectrum disorder	9 (3.8)
Disturbance of stereotyped movement	9 (3.8)
Autism spectrum disorder	4 (1.7)
Major neurocognitive disorder	4 (1.7)
Attention deficit/Hyperactivity disorder	2 (0.9)
Another medical condition that has lasted for more than 3 months	
Yes	71 (30.3)
**Number of different prescribed medicines per day** [*M* (±*DP*; min-max)]	3.98 (3.21; 0–16)
**Polypharmacy**	
Does not take any medication	37 (15.8)
Not polymedicated (< 5 medications/day)	136 (58.1)
Polymedicated (≥5 medications/day)	61 (26.1)
Hospital admissions over the past year^*^ [*M* (±*DP*; min-max)]	0.09 (0.35; 0–2)
Yes	18 (7.7)
No	215 (92.3)
Visits to emergency service in the past year^*^ [*M* (±*DP*; min-max)]	0.33 (0.07; 0–7)
Yes	53 (22.7)
No	180 (77.3)
**Consults with the family doctor in the past year** [*M* (±*DP*; min-max)]	1.34 (1.56; 0–12)
No	62 (26.6)
Yes	171 (73.4)
**Malnutrition risk**	
Malnourished	11 (4.7)
At risk of malnutrition	105 (44.9)
Normal	118 (50.4)
**BMI (kg/m** ^2^ **)** ^**^	
Underweight (< 18.5)	4 (1.7)
Normal weight (18.5–24.9)	58 (24.8)
Overweight (25.0–29.9)	92 (39.3)
Obesity (≥30)	77 (32.9)
BMI [M (±DP; min-max)]	28.59 (6.26; 17.36–77.48)

^*^One participant was excluded due to an outlier in the mean (>3 SD), in accordance with the criteria for detecting extreme values; ^**^three participants with no information on weight and height; n, number; M, mean; SD, standard deviation; %, percentage; min, minimum; max, maximum; ID, intellectual disability. Kg, kilograms; m, meters.

In terms of malnutrition risk, 50.4% had a normal nutritional status (Table 3). However, 105 participants (44.9%) were at malnutritional risk, and 4.7% were malnourished. In terms of BMI, there was a high prevalence of overweight (*n* = 92; 39.3%) and obesity (77; 32.9%). The mean BMI values were 28.59 ± 6.26 kg/m^2^.

**Table 3:** Characterization of activities of daily living (*n* = 234).

**Variables**	*n* (%)
**BADL—*Barthel Index*** *M* (±*DP*; min-max)	77.67 (26.56; 0–100)
Total dependency	17 (7.3)
Severe dependency	37 (15.8)
Moderate dependency	84 (35.9)
Mild dependency	22 (9.4)
Independent	74 (31.6)
**Feeding**	
Independent	140 (59.8)
Needs some help	67 (28.6)
Dependent	27 (11.5)
**Transfers (bed to chair and back)**	
Independent	175 (74.8)
Needs some help	33 (14.1)
Needs help from another person	1 (0.4)
Dependent	25 (10.7)
**Personal hygiene (Toileting)**	
Independent	142 (60.7)
Dependent	92 (39.3)
**Toilet use**	
Independent	157 (67.1)
With help	51 (21.8)
Dependent	26 (11.1)
**Bathing**	
Independent	119 (50.9)
Dependent	115 (49.1)
**Mobility**	
Independent	143 (61.1)
With some help	45 (19.2)
Independent in a wheelchair	39 (16.7)
Immobile	7 (3)
**Stairs**	
Independent	154 (65.8)
Needs help	45 (19.2)
Dependent	35 (15)
**Dressing**	
Independent	141 (60.3)
Needs help	75 (32.1)
Dependent	18 (7.7)
**Bowel control**	
Continent	190 (81.2)
Occasional accidents	30 (12.8)
Incontinent	14 (6)
**Bladder control**	
Continent	173 (73.9)
Occasional accidents	46 (19.7)
Incontinent	15 (6.4)
**IADL—Index of** ***Lawton** M* (±*DP*; min–max)	26.7 (6.35; 9–30)
Independent	0 (–)
Severely dependent	200 (85.5)
Moderately dependent	34 (14.5)
**Housekeeping**	
Maintains house alone	10 (4.3)
Does everything except heavy lifting	20 (8.5)
Performs light daily	94 (40.2)
Needs help with all tasks	41 (17.5)
Unable to perform any task	69 (29.5)
**Laundry**	
Does personal laundry	13 (5.6)
Launders small items	33 (14.1)
Unable	188 (80.3)
**Food preparation**	
Plans, prepares and serves independently	5 (2.1)
Prepares if supplied with ingredients	20 (8.5)
Prepares ready meals	31 (13.2)
Unable	178 (76.1)
**Shopping**	
Shops independently	11 (4.7)
Only does little shopping	40 (17.1)
Accompanied	70 (29.9)
Unable	113 (48.3)
**Use of the telephone**	
Uses it without difficulty	47 (20.1)
Dials a few familiar numbers	37 (15.8)
Needs help	69 (29.5)
Unable	81 (34.6)
**Transportation**	
Travels on public transportation or drives own car	54 (23.1)
Only travels via taxi	1 (0.4)
Needs to be accompanied	90 (38.5)
Unable	89 (38)
**Finances**	
Manages financial matters independently	9 (3.8)
Manages day-to-day purchases	83 (35.5)
Unable	142 (60.7)
**Medication**	
Responsible for taking medication	25 (10.7)
Responsible if medication is prepared	81 (34.6)
Unable	128 (54.7)

BADLs, basic activities of daily living; IADLs, instrumental activities of daily living: n, number; M, mean; SD, standard deviation; %, percentage; min, minimum; max, maximum.

### Functionality

Regarding BADLs (Table 3), 96 participants (41%) were independent or had mild dependence, 84 (35.9%) had moderate dependence, and 54 (23.1%) had total or severe dependence. In all activities, the majority of the participants (>50%) were independent. The tasks involving the highest number of dependent participants were bathing (*n* = 115; 49.1%) and personal hygiene (toileting; *n* = 92; 39.3%). The tasks with the highest number of independent participants were transfers (*n* = 175; 74.8%), using the toilet (*n* = 157; 67.1%), going up and down stairs (*n* = 154; 65.8%), and mobility (*n* = 143; 61.1%).

In terms of IADLs (Table 3), the results indicate that there were no independent participants. The majority (*n* = 200; 85.5%) were severely dependent, and 34 (14.5%) were moderately dependent. Most of the participants were unable to perform the following instrumental activities: laundry (*n* = 188; 80.3%), food preparation (*n* = 178; 76.1%), handling of finances (*n* = 142; 60.7%) and responsibility for own medications (*n* = 128; 54.7%).

### Frailty

Based on the four levels of frailty (Table 4), 109 participants (48%) were classified as robust, followed by 103 (45.4%), who were classified as frail, of whom two participants were in the terminal stage. Fifty-three participants were assessed according to the stage of dementia, with 32 classified as mild (level 5 on the frailty scale), 13 classified as moderate (level 6), six classified as severe (level 7), and two classified as very severe (level 8). Using a cut-off point of 5 on the clinical frailty scale, 122 participants (53.7%) were classified as “non-frail” and 105 (46.3%) as “frail.”

**Table 4:** Characterization of frailty (Clinical Frailty Scale) (*n* = 227^*^).

**Frailty**	*n* (%)
Robust (1–3)	109 (48)
Pre-frailty (4)	13 (5.7)
Frail (5–7)	103 (45.4)
Terminal stage (8–9)	2 (0.9)
**Frailty (** ***n*** **, %)**	
Non-frail (< 5)	122 (53.7)
Frail (≥ 5)	105 (46.3)

^*^7 missing values; n, number; %, percentage.

### Frail vs. non-frail and the association between sociodemographic, clinical, and functional variables

The results of the association between sociodemographic and clinical variables and “non-frail” vs. “frail” status show significant differences in the variables described in Table 5.

**Table 5:** Frail vs. non-frail: association between sociodemographic and clinical variables and functional status.

Variables	Non-frail	Frail	Statistical results
	***n*** **(%)**	***n*** **(%)**	
	122 (52.1)	105 (44.9)	
**Sociodemographic**			
**Sex**			
Male	79 (61.7)	49 (38.3)	χ^2^ = 7.507; *p* = 0.007 | OR = 2.1, [1.23–3.58]
Female	43 (43.4)	56 (56.6)	
**Can read and write**		
No	71 (47.3)	79 (52.7)	χ^2^ = 7.311; *p* = 0.007 | OR = 2.18, [1.23–3.86]
Yes	51 (66.2)	26 (33.8)	
**Years of formal education**			
< 4 years	32 (48.5)	34 (51.5)	χ^2^ = 11.071; *p* = 0.004 | *V* = 0.221 [0.11–0.34]
4 years	54 (47.8)	59 (52.2)	
5–12 years	36 (75)	12 (25)	
**Living in**			
Institution	36 (42.9)	48 (57.1)	χ^2^ = 4.247; *p* = 0.039 | OR=1.79 [1.03–3.11]
Community	75 (57.3)	56 (42.7)	
**ID degree**			
Mild	23 (74.2)	8 (25.8)	χ^2^ = 17.364; *p* = 0.001 | *V* = 0.277 [0.18–0.39]
Moderate	70 (58.3)	50 (41.7)	
Severe	26 (44.1)	33 (55.9)	
Profound	3 (17.6)	14 (82.4)	
**Malnutrition risk**			
Malnourished	0 (–)	11 (100)	χ^2^ = 81.409; *p* < 0.001 | *V* = 0.599 [0.50–0.70]
At risk of malnutrition	28 (27.2)	75 (72.8)	
Normal	94 (83.2)	19 (16.8)	
**Another medical condition that has lasted for more than 3 months**			
No	91 (58.3)	65 (41.7)	χ^2^ = 4.225; *p* = 0.04 | OR=1.81 [1.02–3.18]
Yes	31 (43.7)	40 (56.3)	
**Polypharmacy**		
No medication intake	26 (74.3)	9 (25.7)	χ^2^ = 7.918; *p* = 0.019 | *V* = 0.187 [0.08–0.31]
1–4 medicines	70 (52.2)	64 (47.8)	
≥5 medicines	26 (44.8)	32 (55.2)	
**Continuous variables**	***M*** ±***DP***	***M*** ±***DP***	**Statistical results | Effect (CI 95%)**
Age	52.61 ± 5.79	54.25 ± 7.11	*U* = 5,659; *p* = 0.13 | *r* = 0112 [−0.03, 0.26]
BMI^***^	27.98 ± 4.86	29.20 ± 07.59	*U* = 5,694; *p* = 0.27 | *r* = 0.086 [−0.07, 0.23]
BADL	86.43 ± 18.59	66.90 ± 30.80	*U* = 3,916.5; *p* < 0.001| *r* = −0.39[−0.51, −0.25]
IADL	24.78 ± 6.90	29.03 ± 4.87	*U* = 4,046.5; *p* < 0.001 | *r* = 0.368 [0.23, 0.49]
Medicines per day	3.51 ± 3.12	4.52 ± 3.23	*U* = 5,227; *p* = 0.016 | *r* = 0.184 [0.04, 0.33]
Hospital admission over the past year	0.05 ± 0.22	0.15 ± 0.46	*U* = 5,912; *p* = 0.06 | *r* = 0.068 [−0.08, 0.22]
Emergency visits in the past year^a^	0.33 ± 0.87	0.36 ± 0.67	*U* = 5,964; *p* = 0.303 | *r* = 0.06 [−0.09, 0.21]
Consults with the family doctor in the past year^a^	1.18 ± 1.40	1.46 ± 1.41	*U* = 5,538; *p* = 0.084 | *r* = 0.127 [−0.02, 0.27]

^*^7 missing values; ^**^12 participants were excluded due to missing values and ambiguous living situations (“others” and “rotation”); ^***^3 participants were excluded due to no information on weight and height; ^*a*^one extreme outlier removed from analysis; n, number; M, mean; SD, standard deviation; %, percentage; min, minimum; max, maximum; χ^2^, chi-square; U, Mann–Whitney U-test; p, significance level; OR, odds ratio; CI, confidence interval; BADL, basic activities of daily living; IADL, instrumental activities of daily living; ID, intellectual disability; r, rank-biserial correlation.

Frailty is more common among (i) women (χ^2^ = 7.507; *p* = 0.007, and OR = 2.1 [95% CI 1.23–3.58]), (ii) participants who could not read or write (χ^2^ = 7.31, *p* = 0.007, and OR = 2.18 [95% CI 1.23–3.86]), (iii) those who had not completed primary education (χ^2^ = 11.07, *p* = 0.004, and Cramér's V [V] = 0.22), and (iv) those with more severe degrees of intellectual disability (χ^2^ = 17.36, *p* = 0.001, and *V* = 0.28), and (v) institutionalized participants compared with those living in the community (χ^2^ = 4.25, *p* = 0.039, OR = 1.79 [95% CI 1.03–3.11], and *n* = 215). Malnutrition risk was strongly associated with frailty (χ^2^ = 81.41, *p* < 0.001, and *V* = 0.60). All participants classified as malnourished were frail, whereas those with normal nutritional status showed a markedly lower prevalence of frailty. It was observed that all participants who were malnourished were frail, while those with a normal nutritional status had a lower prevalence of frailty. The presence of medical conditions lasting longer than three months was also associated with frailty (χ^2^ = 4.23, *p* = 0.04, and OR = 1.81 [95% CI 1.02–3.18]). Polypharmacy was more common among frail individuals, while a higher proportion of non-frail participants did not take medication (χ^2^ = 7.92, *p* = 0.019, and *V* = 0.19). In addition, the number of medications also differed between the groups (*U* = 5,227, *p* = 0.016, and *r* = 0.18 [95% CI 0.04–0.33]), being higher among frail (4.52 ± 3.23) than non-frail participants (3.51 ± 3.12). In ADLs, statistically significant differences were observed between frail and non-frail participants, both in BADLs (*U* = 3,916.5; *p* < 0.001; *U* = 3,916.5, *r* = −0.39 [95% CI −0.51 to −0.25]) and in IADLs (*U* = 4,046.5; *p* < 0.001, *r* = 0.368 [95% CI 0.23–0.49]). Frail participants showed greater dependence in BADLs (66.90 ± 30.80 vs. 86.43 ± 18.59) and IADLs (29.03 ± 4.87 vs. 24.78 ± 6.90) compared with non-frail individuals.

## Discussion

This study focused on the characterization of people with ID aged ≥ 45 years in terms of clinical conditions, functionality and frailty. The results show that people with ID, as they age, present a unique and complex clinical condition demanding an increased intensity of support.

The participants had a mean age of 53.32 years, ranging from 45 to 77 years, reflecting the increase in life expectancy observed internationally in this population group (El Mrayyan et al., 2025). Almost all the participants were single (97.9%) and childless (98.3%) and were living with relatives (52.6%) or in social care residences (39.3%). They had low levels of academic qualifications, with 66.2% unable to read or write. In addition, 89.3% were retired, and 82.5% relied on social security benefits or pensions as their main source of income. These findings are consistent with other studies (e.g., Ortega-Camarero et al., 2021), reinforcing the lack of access to paid employment and to income of their own. These characteristics show a vulnerable group throughout life, with aging adding more vulnerability and pointing to limited social participation, one of the determinants of healthy aging, which includes difficulties in building a social network of friends and peers that extends beyond the family and the institution (staff and peers) in which they are supported or live. Many of the older adults with ID in this study had been supported by organizations since childhood and had been with their current organization for an average of 26.32 years (Harrison et al., 2021; McCausland et al., 2021). Institutional longevity seems to be relevant to establishing stability, a sense of protection and attachment. Nevertheless, it can also function as a restraint to being open to other networks and experiences.

### Aging with ID: a unique and complex clinical conditions

Among the participants, 67.1% had a mild to moderate degree of ID, while 32.9% had a severe to profound degree of ID. The life expectancy of people with ID has been linked to the severity of their ID. It is estimated that people with mild ID have an average life expectancy similar to that of the general population; for people with moderate ID, life expectancy is estimated to be 65–70 years; in the case of severe and profound ID, life expectancy is about 50 years (Bittles et al., 2002; Patja et al., 2000; Srasuebkul et al., 2026). However, it is important to consider the presence of comorbidities, as these can significantly influence longevity.

In this study, older adults with ID had various comorbidities, such as epilepsy (22.6%), dementia (22.6%), impulse control disorder (19.7%), and anxiety disorder (15.4%). For example, epilepsy increases the risk of death in all people with ID, regardless of the severity of the condition (Patja et al., 2000). Dementia is particularly common in people with trisomy 21 (Dolan et al., 2019). In addition, mental health problems (e.g., anxiety, depression and bipolar disorder) are common among older adults with ID, even more than among the general population (Grohmann et al., 2024). Our sample also included participants with high blood pressure, heart problems, type 2 diabetes, cancer, respiratory problems, high cholesterol, and thyroid problems. Furthermore, 44.9% were at risk of malnutrition, and 32.9% were classified as obese (a relevant nutritional paradox), with pre-obesity and obesity constituting a risk factor for multiple diseases (e.g., diabetes and cardiovascular disease). Chronic conditions and multimorbidity make older adults more likely to be on multiple medications, to be hospitalized and to make more frequent visits to emergency services (Walsh et al., 2025). Among the participants, 26.1% were on polypharmacy; in the past year, 7.7% had been hospitalized, and 22.7% had visited emergency services at least once. Older adults with ID are among the heaviest users of healthcare services, being twice as likely to visit emergency services and be admitted to hospital compared with adults without this disability (McKenzie et al., 2016). The literature recommends the implementation of measures and programmes to promote health and prevent chronic diseases among this population (Rutherford et al., 2024). Overall, older adults with ID present a complex and unique clinical condition (McKenzie et al., 2016) in which care must be adapted (Sheerin et al., 2025). The unique nature of the aging process of persons with ID needs to be addressed. They often have chronic conditions throughout life, and it is the deterioration, rather than the presence, that indicates a decline—that is, the onset of the complexities associated with aging (McKenzie et al., 2016). For this reason, signaling the onset of frailty is key to punctuating the aging complexities in this population group.

Considering our results on frailty, we can note that 48% (the robust) have (probably) not yet entered the complexity that marks the onset of aging, 5.7% (pre-frail) are at the beginning of this stage, 45.4% (the frail) are experiencing the complexity associated with aging, and 0.9% are at the end of life. The literature suggests that frailty can affect individuals with ID, on average, 20–30 years earlier than expected in the general population (Grohmann et al., 2024). This population must be assessed for frailty at an earlier age using validated instruments. Although the CFS has been used to assess frailty in older adults with ID (Grohmann et al., 2024), several instruments have been specifically developed and validated to evaluate frailty in this population. These include the Intellectual Disability-Frailty Index and its short form (Schoufour et al., 2013), the Vienna Frailty Questionnaire for Persons with Intellectual Disabilities (Brehmer-Rinderer et al., 2013), and the IDS-TILDA Frailty Index (McFeely et al., 2024). However, none of these instruments has yet been validated for the Portuguese population and is therefore not used in this study. Future studies could validate these instruments for the Portuguese older population with ID to help more accurately identify the onset of frailty in these persons, thereby enabling appropriate healthy aging interventions.

In addition, frailty in people with ID predicts some adverse outcomes, including reduced mobility, an increase in falls, greater use of medication and more hospital admissions (McKenzie et al., 2016). Our results suggest that frailty is more common among women, those who cannot read or write or who have not completed primary education, and participants with severe ID. Therefore, particular attention should be paid to this group when planning healthy aging interventions. The results also show that the malnourished participants were frail, while those with a normal nutritional status had a lower prevalence of frailty. Therefore, nutritional care is essential for this group, and the involvement of nutritionists in their care should be demanded. Moreover, our results show that the presence of medical conditions lasting longer than 3 months, polypharmacy and the number of medications are significantly higher among frail individuals than among non-frail individuals. These may be associated to the onset of the complexity associated with aging and involving a higher intensity of care.

### Aging with ID: an increase in support intensity

This unique and complex clinical condition requires an increase in support intensity for people with ID as they age (Di Giacomo et al., 2025). In this study, around a third of the participants were independent in BADLs, a significant proportion was completely or severely dependent, and the majority were severely dependent on IADLs. Moreover, participants classified as frail showed greater dependence in BADLs and IADLs. However, this association should be interpreted with caution, since the CFS includes information on functionality, mobility, cognition, and dependence. Therefore, the association between CFS-defined frailty and BADL/IADL may partly reflect overlap between the constructs being measured, rather than an independent confirmation of frailty status. Frailty and associated levels of dependency put additional physical and emotional strain on informal and formal carers. Informal carers are usually the parents who are old and getting older (Chou and Kröger, 2022). Formal carers, usually the workforce providing social care in services for people with ID in Portugal, are usually aged ≥50 years, which may limit their ability to cope with the intensity of care required (Letra et al., 2025; Barbosa et al., 2016). Physical strain may be exacerbated by the prevalence of excess weight and obesity combined with dependence on many ADLs. Emotional strain may be linked to caring for people with multiple health problems, including mental health issues, along with challenging behaviors. Some aspects related to BADLs and IADLs need particular attention. The literature indicates that the total score for BADLs tends to be determined by mobility, while the total score for IADLs is determined by the degree of ID (Hilgenkamp et al., 2011). In this study, 61.1% of the participants were independent in terms of mobility. Thus, it is important to promote mobility among older adults with ID to maximize their independence (Hilgenkamp et al., 2011). In addition, functioning in IADLs is associated with physical, cognitive and perceptual abilities, which can be influenced by training and support (Hilgenkamp et al., 2011). Functioning in ADLs declines with age in the general population and in people with ID, but this decline seems to occur earlier in people with ID. It should also be noted that people with ID already have some pre-existing dependency, which worsens with aging (Hilgenkamp et al., 2011). More research is needed to fully address the profiles of older adults with ID. The results show the vulnerability of this group, which demands specific healthy aging interventions.

### Limitations and research perspectives

This study has some limitations. The sample was not representative, although it was a robust exploratory sample. A representative sample would allow for generalizations, which is not possible in this study. Future studies could seek representative samples and include more people with ID aged ≥ 65 years, enabling an analysis of the profiles at a more advanced stage of aging. In addition, the low response rate, together with the lack of organization recruitment data, limited the assessment of potential selection bias, cluster sizes, participant distribution across organizations, organizational-level representativeness, and clustering by organization or mediator. Furthermore, as data anonymization procedures prevented linking responses to a specific mediator, it was not possible to assess inter-rater reliability, which may represent an additional source of bias. The instruments used in this study have been validated for the older Portuguese population and have been used in Portugal and other countries with older adults with ID. However, they have not been validated for Portuguese older adults with ID, which may be a source of bias in the results. Thus, it is recommended that future studies focus on validating instruments for older people with ID in Portugal. Frailty is a significant factor in the occurrence of falls (Yang et al., 2023). In the sample study, a significant prevalence of frailty was observed, as well as the presence of polypharmacy, dependency and multimorbidity, all of which are recognized as factors contributing to the occurrence of falls. This study did not include questions on falls; thus, it is recommended that future studies take these into account. Moreover, results should be interpreted considering the cross-sectional design and the convenience sample. The analyses were exploratory and based mainly on univariable comparisons, without adjustment for multiple testing or multivariable modeling. Future studies should use multivariable approaches to examine the independent contribution of sociodemographic, clinical and functional variables to frailty.

## Conclusion

Older adults with ID represent one of the most clinically complex and socially vulnerable groups in aging societies. However, they remain among the least visible in health and social policies. This study suggests that aging with ID is more than a gradual extension of a lifelong condition. It is a unique and complex compounding process that comprises comorbidities, frailty, deepens dependence and intensifies care needs at a pace and scale that current systems seem ill-equipped to meet.

The results are clear: Nearly half of the participants were frail, the majority were severely dependent in IADLs, more than one third were taking multiple medications, and malnutrition risk coexisted with obesity in a nutritional paradox. Older adults with ID require a high support intensity and tailored healthy aging interventions that should anticipate earlier frailty onset, promote functional independence and mobility, ensure nutritional care and draw on multidisciplinary teams. As life expectancy in this population continues to rise, the question is no longer whether to act but whether the systems in place will be ready. This study offers both evidence and the imperative to begin.

## Data Availability

The datasets analyzed during the current study are not publicly available due to institutional policy, but are available from the corresponding author upon reasonable request. Requests to access these datasets should be directed to lilianax@ua.pt.
